# Avian agnosia: A window into auditory semantics

**DOI:** 10.1016/j.neuropsychologia.2019.107219

**Published:** 2019-11

**Authors:** J.A. Mole, I.W. Baker, J.M. Ottley Munoz, M. Danby, J.D. Warren, C.R. Butler

**Affiliations:** a Russell Cairns Unit, John Radcliffe Hospital, Oxford, UK; b Department of Neuropsychology, National Hospital for Neurology and Neurosurgery, London, UK; c Dementia Research Centre, UCL Institute of Neurology, University College London, London, UK; d Nuffield Department of Clinical Neurosciences, University of Oxford, UK

**Keywords:** Bird, Auditory agnosia, Semantic dementia, Expertise

## Abstract

The functional and neural organisation of auditory knowledge is relatively poorly understood. The breakdown of conceptual knowledge in semantic dementia has revealed that pre-morbid expertise influences the extent to which knowledge is differentiated. Whether this principle applies to a similar extent in the auditory domain is not yet known. Previous reports of patients with impaired auditory vs. intact visual expert knowledge suggest that expertise may have differential effects upon the organisation of auditory and visual knowledge. An equally plausible alternative, however, is that auditory knowledge is simply more vulnerable to deterioration. Thus, expertise effects in the auditory domain may not yet have been observed because knowledge of auditory expert vs. non-expert knowledge has yet to be compared. We had the opportunity to address this issue by studying SA, a patient with semantic dementia and extensive pre-morbid knowledge of birds. We undertook a systematic investigation of SA's auditory vs. visual knowledge from matched expert vs. non-expert categories. Relative to a group of 10 age, education and IQ matched bird experts, SA showed impaired auditory vs. intact visual avian knowledge, despite intact basic auditory perceptual abilities. This was explained by independent effects of modality and expertise. Thus, he was also disproportionately impaired for auditory vs. visual knowledge of items from non-expert categories. In both auditory and visual modalities, his performance was relatively more impaired on tests of non-expert vs. expert knowledge. These findings suggest that, while auditory knowledge may be more vulnerable to deterioration, expertise modulates visual and auditory knowledge to a similar extent.

## Highlights

• Selective preservation of knowledge in a bird expert with semantic dementia.
• Impaired auditory vs. intact visual avian knowledge.
• However, performance better for auditory and visual expert vs. non-expert knowledge.
• First demonstration that expertise protects against loss of auditory knowledge.

## 1 Introduction

Over the past few decades significant progress has been made in furthering our understanding of the neural organisation of conceptual knowledge. Largely for methodological reasons, this has focused on verbal and visual materials. An equivalent understanding of auditory knowledge is lacking and, even to this date, its characterisation remains a relatively unchartered frontier.

Patients with ‘semantic dementia’ (SD) have played a critical role in furthering understanding of the organisation of semantic knowledge (Patterson and Lambon Ralph, 2016; Snowden et al., 1989; Warrington, 1975). Such patients have atrophy and hypometabolism of the inferior aspects of the anterior temporal lobes (ATL) that is bilateral, although typically asymmetric (Hodges and Patterson, 2007). While performance in many cognitive faculties remains relatively spared, SD patients exhibit a progressive deterioration of semantic knowledge (Adlam et al., 2009; Hodges et al., 1992; Warrington, 1975). Patterns of impairment are remarkably consistent and have revealed key insights into the organisational structure of concepts in memory. For example, double dissociations between impairments in verbal vs. visual knowledge in SD and other conditions suggest that knowledge has, in these modalities at least, cognitive and neural substrates that are to some extent separable (McCarthy and Warrington, 1988; Snowden et al., 2018; Snowden et al., 2004; Warrington and McCarthy, 1994). More recently, it has become apparent that patients with SD who had pre-morbid expertise with specific categories of objects show relatively intact knowledge of items from their expert category (Jefferies et al., 2011; Robinson and Cipolotti, 2001). For example, Jefferies et al. (2011) reported on a detailed investigation of two patients with SD: a former automotive worker and former botanist. Semantic knowledge of expert and non-expert categories were assessed using picture naming and picture-word matching tasks. Whilst both patients showed the typical pattern of an inability to differentiate between highly similar concepts from their non-expert categories, the former automotive worker showed selective preservation of car knowledge and the former botanist showed selective preservation of information about plants, when compared against non-experts in their respective fields. These findings have important implications for understanding the organisation of conceptual knowledge, as they reveal that expertise influences the extent to which knowledge is differentiated within the healthy brain.

Whether similar principles underlie the organisation of auditory knowledge is less clear. Although visual and auditory knowledge may be lost in tandem (Gainotti, 2013), recently reported dissociations suggest that knowledge in these domains is to some extent modality specific (Hailstone et al., 2010; Luzzi et al., 2018). Remarkably, what is not yet known, is whether expertise effects visual and auditory knowledge to a similar extent. Two cases of SD have been reported with loss of auditory knowledge for their expert categories. While this may suggest that expertise does not protect against deterioration of auditory representations, other interpretations remain equally plausible. First, Omar et al. (2010) documented a professional trumpeter with SD, who retained the ability to recognise musical notation but showed an impairment in auditory music knowledge. Second, Muhammed et al. (2018) reported on BA, a case of SD who had premorbid expertise in the domain of bird knowledge. Muhammed et al. (2018) tested BA's knowledge of avian characteristics (size, behaviour and habitat) in the verbal, visual and auditory modalities. Relative to the performance of three healthy bird experts of a similar age, BA had an impairment in verbal and auditory bird knowledge but relatively preserved knowledge of avian visual attributes. There was, however, more complexity to this pattern. For both auditory and visual stimuli, BA had unimpaired knowledge of size and migratory behaviour and impaired knowledge of habitat, suggesting that the dissociation between auditory and visual knowledge was less than clear cut. Crucially, in neither the study by Omar et al. (2010) or Muhammed et al. (2018) was a comparison made between knowledge of auditory objects from comparable expert vs. non-expert categories. Although Muhammed et al. (2018) compared BA's knowledge of birds and famous people, as the authors acknowledged, it was not possible to draw meaningful comparisons between performance on these tasks, as there were important differences in task requirements and these categories require different levels of discrimination. There are two plausible interpretations that could explain the difference between performance in visual vs. auditory knowledge. First, expertise may have differential effects upon the organisation of auditory and visual knowledge. Second, auditory knowledge may simply be more vulnerable to deterioration and so expertise effects in the auditory domain were not obvious because auditory expert vs. non-expert knowledge was not compared. Thus, a key question remains unanswered: does expertise modulate auditory and visual knowledge to a similar extent?

We were presented with a rare opportunity to address this question when SA, a patient with SD and premorbid expertise in bird ringing^1^ presented to our clinic. Bird knowledge represents a single semantic domain that is comparably accessible via the auditory and visual sensory modalities and is a category with which some individuals acquire extensive expertise (typically across verbal, visual and auditory modalities). In an attempt to distinguish between effects of modality and expertise, we carefully designed a battery of tests of visual and auditory knowledge from expert and non-expert categories.

## 2 Material and methods

### 2.1 Participants

**Fig. 1:**
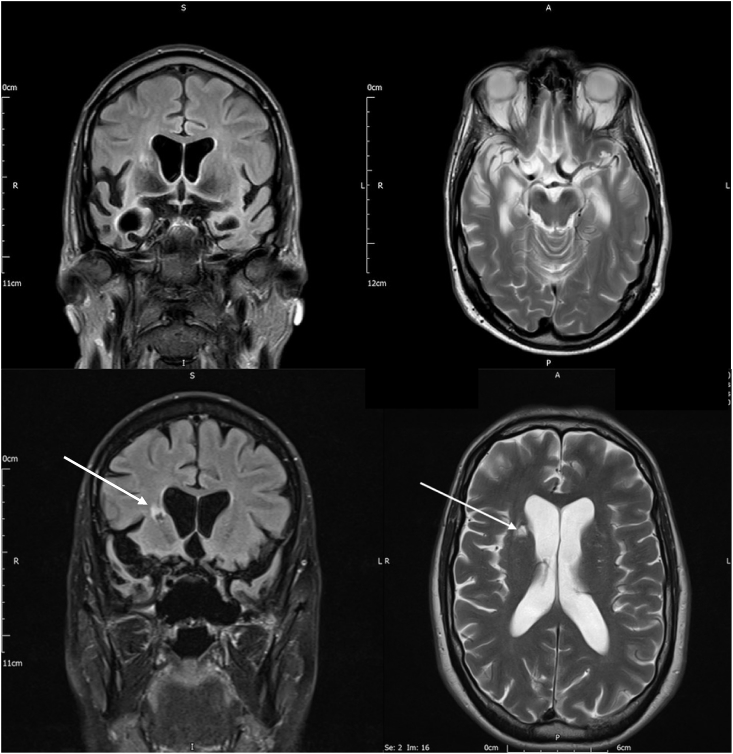
Magnetic Resonance Imaging scan of patient SA. This was reported as showing bilateral anterior temporal lobe atrophy, worse on the right than the left. A small, old lacunar infarct in the right putamen was also noted (indicated by arrows).

SA, a 61-year-old recreational bird ringer with a diagnosis of SD, participated in this study. He has a degree in Geology and formally worked for a company that managed geographical and environmental testing data. At the age of 10, he developed what was to become a life-long interest in bird ringing and from 2006 he ran a local bird ringing project. In 2012 his company went into bankruptcy and he became profoundly low in mood. He was treated with citalopram and later venlafaxine but with no beneficial effects. In January 2016 he failed to complete his annual bird ringing report, which he attributed to demotivation. In July of that year he had an episode of slurred speech and poor co-ordination lasting a couple of minutes. Structural magnetic resonance brain imaging performed at this time showed marked involutional changes in the anterior poles of both temporal lobes, more marked on right, and an established haemorrhagic stroke in the right putamen (see Fig. 1). In August 2016 he was assessed at his local memory clinic, where he reported a gradual deterioration over the previous year in his recognition of birds and his ability to recognise friends and family. He was referred on to the Oxford Cognitive Disorders Clinic, where he was seen in February 2017. He again emphasised his difficulty with bird recognition but stated that this problem applied specifically to identifying birds by their calls, rather than visually. He also acknowledged difficulties recognising people's faces, either people he knew personally or celebrities on the television, and identifying people's voices on the telephone. His wife noted some gaps in his remote memory, for example, occasions when he did not recognise a place that he had visited previously. He described a marked “lack of energy” and his wife reported that he had become routine-bound and inflexible, lacking in empathy and increasingly limited in his interests, now restricted almost entirely to birds. She noted that he had started to eat more sweet foods, which was a noticeable change in his food preference. However, he was completely independent in all activities of daily living and was still driving with no apparent difficulties. During the interview, his spoken language was intact in conversation but his speech was slow and aprosodic. He occasionally gave tangential answers to questions and made inappropriate interruptions during the interview. He scored 86/100 on the Addenbrooke's Cognitive Examination – Third edition (Hsieh et al., 2013), (subscores: attention = 18/18, memory = 16/26, fluency = 11/14, language = 25/26 and visuospatial = 16/16). Of note, he was unable to identify a picture of a camel and, although his repetition of proverbs was intact, his interpretations were concrete. For example, when asked what ‘All that glitters is not gold’ means, he said ‘well, there are lots of things other than gold that glitter – for example, silver and diamonds’.

We recruited eleven local male control subjects with expertise in bird ringing via the Oxford Ornithological Society (OOS) and the British Trust for Ornithology's (BTO) Ringing Scheme. Bird ringers, rather than general ornithologists, were recruited as SA's expertise were specifically in bird ringing. All participants were healthy and had normal visual and auditory acuity, except for one participant who was excluded due to self-reported auditory difficulties. The remaining ten participants were well-matched to SA in terms of age, years of education and number of years of experience bird ringing (see Table 2)^2^

Control participants were tested at the Russell Cairns Unit, John Radcliffe Hospital in Oxford. SA's testing was split across three sessions to avoid fatigue. The first testing session was conducted at the Russell Cairns Unit and the second two at his home. Informed consent was obtained prior to testing. Ethical approval was received from South Central Oxford Research Ethics Committee (REC no: 08/H0606/133).

### 2.2 Background neuropsychological assessment

SA and control participants were administered the Wechsler Adult Intelligence Scale – Fourth edition (WAIS-IV; Wechsler, 2008) Vocabulary and Matrix Reasoning subtests in April 2017. SA agreed to complete the experimental investigations but declined further testing using standard neuropsychological tests. The results from his neuropsychological assessment at his local memory clinic (August 2016) and his performance on WAIS-IV Vocabulary and Matrix Reasoning subtests (April 2017) are presented in Table 1.

**Table 1:** SA's performance on standardised neuropsychological tests.

	Score	Scaled Score/Percentile
Optimum Functioning		
National Adult Reading Test	35	Estimated FSIQ = 112
Intellectual Functioning		
WAIS-IV Vocabulary*	33	Scaled score = 9
WAIS-IV Matrix Reasoning*	21	Scaled score = 14
Verbal Memory		
WMS-IV Logical Memory I	27	Scaled score = 11
WMS-IV Logical Memory II	26	Scaled score = 12
HVLT-R Immediate Recall	24	16 - 25th percentile
HVLT-R Discrimination Index	9	16th percentile
HVLT-R Delayed recall	4	<1st percentile
Visual Memory		
WMS-IV Visual Reproduction I	39	Scaled score = 12
WMS-IV Visual Reproduction II	21	Scaled score = 10
Attention		
D-KEFS Trails 1	24″	Scaled score = 10
Executive Functioning		
D-KEFS Trails 4 (1 error)	99″	Scaled score = 10
D-KEFS Letter Fluency	37	Scaled score = 10
MEAMS Motor Perseveration subtest	5/5	
Clock Drawing	4/4	
Weigl Sorting test	4/4	
Language/Semantic Memory		
D-KEFS Category Fluency	38	Scaled score = 10
Graded Naming Test	16	Scaled score = 7

DKEFS = Delis-Kaplan Executive Function System (Delis et al., 2001), HVLT-R = Hopkins Verbal Learning Test – Revised (Benedict et al., 1998), FSIQ = Full scale intelligence quotient, Graded Naming Test (McKenna and Warrington, 1983), MEAMS Motor Perseveration subtest (Golding, 1988), National Adult Reading Test (Nelson and Willison, 1992), Weigl Sorting test (Weigl, 1941), WAIS-IV = Wechsler Adult Intelligence Scale – Fourth edition (Wechsler, 2008), WMS-III = Wechsler Memory Scale – 3rd Edition (Wechsler, 1997). *conducted in April 2017.

**Table 2:** Demographics and intellectual functioning.

	Control Mean	Control SD	SA	*t*	*p*
Age	66.80	10.26	61	-0.54	0.60
Years of education	15.90	6.57	16	0.02	0.99
Years of expertise with birds	50.10	7.23	51	0.12	0.91
WAIS-IV- Vocabulary	27.80	3.85	33	1.29	0.23
WAIS-IV- Matrix Reasoning	19.80	3.26	21	0.35	0.73

*p* values are two-tailed. SD = standard deviation. WAIS-IV= Wechsler Adult Intelligence Scale – Fourth edition (Wechsler, 2008).

### 2.3 Experimental investigation

We administered a range of tests of visual and auditory expert and non-expert knowledge.

With the exception of the auditory perceptual discrimination task and the bird gender discrimination task, all tasks required participants to respond by naming stimuli. Thus, demands were similar across different tasks. This was feasible as SA demonstrated good verbal knowledge of birds in general conversation (he was able to discuss birds and described his previous ringing reports in detail). For all tests, participants could view images for as long as they liked and listen to sounds as many times as they wished. Visual stimuli were cropped to remove background clues and, unless otherwise specified, all images were presented in colour. Images were manipulated to be of equal size, to increase the difficulty of the test, and this was explained to the participants.

#### 2.3.1 Basic auditory perception

##### 2.3.1.1 Auditory perceptual discrimination test

A 10-item test of auditory perceptual discrimination was developed. On each trial participants were presented with two bird sounds, one after the other, and were required to indicate whether they were the same bird or different birds. In half of the trials the two bird sounds were identical recordings. Thus, participants' task was to decide if two recordings were identical or not, without any need to consider the type of bird. This test was devised to assess participants’ perceptual abilities to discriminate sounds and did not rely upon semantic knowledge. Audio clips were taken from Sample (2010) and were, on average, 24.48 (*SD* = 9.47) seconds long.

#### 2.3.2 Tests of visual and auditory non-expert knowledge

##### 2.3.2.1 64-Item picture naming task and 13-item auditory naming task

To assess visual knowledge of non-expert categories, we administered the 64-item naming task from the Cambridge Semantic Battery (Adlam et al., 2009). This test requires participants to name black and white line drawings of living and non-living items, presented individually. We also developed a 13-item auditory naming test, by presenting participants with sounds of those non-bird items from the 64-item naming task that have identifiable auditory characteristics. This included four vehicles and nine animals. A complete list of items used is provided in Supplementary Table 1.

##### 2.3.2.2 Insect naming test

To investigate non-expert visual knowledge requiring a similar level of within-category discrimination as that of the bird picture and sound naming tests, we developed a 25-item test of naming single pictures of insects. This was designed to include simple (e.g. ant) and more challenging (e.g. praying mantis) items.

##### 2.3.2.3 Accent naming test

Within-category non-expert auditory knowledge was assessed using a test of accent naming. This comprised nine items, after one item that was failed by all but one control participant was excluded. Participants were awarded one point if they correctly named the country of origin and half a point if their response was an accent that is commonly confused with the correct accent (see Supplementary Table 2 for formal scoring criteria).

##### 2.3.2.4 Famous face and voice naming test

Knowledge of familiar human faces and voices was assessed using a 50-item famous face and voice naming test, adapted from the test reported by Tunnard et al. (In preparation). Twenty-five famous celebrities (five politicians, five actors, five comedians, five singers and five television presenters) were presented once as a face and once as a voice. Faces and voices were intermixed within the same test and presented in a fixed randomised order. The famous individuals included in this test are those who were judged to be highly recognisable and to have achieved fame before the past 5 years (e.g. Margaret Thatcher, Elvis Presley). This was important, given that SA's wife reported that he used to watch television regularly but did this less in more recent years, as his focus of interest was increasingly dominated by birds. A complete list of the famous individuals used is provided in Supplementary Table 3. One control participant was excluded from the analysis, as he reported that he had always been disinterested in popular culture and had always had a poor knowledge of people.

#### 2.3.3 Tests of visual and auditory expert knowledge

##### 2.3.3.1 Bird picture and sound naming test

We investigated SA's visual and auditory knowledge of birds by developing a 50-item bird picture and sound naming test. We included 25 species of bird (five passerines, five wading birds, five owls and birds of prey, five gamebirds and five waterfowl). The annual reports of the local ornithological society and of SA's own records were consulted to guide selection of species that had been sighted in the local area and had been seen by SA in recent years. Each species was presented twice, once as an image and once as a sound. Sounds and images were intermixed within the same test and presented in a fixed semi-randomised order. The image and sound of the same species did not ever appear sequentially.

##### 2.3.3.2 Bird gender discrimination test

As SA reported no decline in his visual recognition of birds, it was anticipated that his performance may be intact on the bird naming task. To be confident that subtle difficulties with visual bird knowledge were not overlooked due to ceiling effects, a more challenging test of bird identification was developed, requiring discrimination of bird gender. Care was taken to select items so that a simple heuristic, such as colour, could not be used to complete the task. For example, to determine correctly the gender of a woodcock (item 2) one must analyse feather shape. In this test, 13 pairs of bird images were presented. On each trial, participants were required to indicate which of the two pictures was the male bird, which appeared on the left seven times and on the right six times in a fixed semi-random order. The maximum score obtainable was thirteen (chance performance = 6.5).

##### 2.3.3.3 Advanced bird naming test

**Fig. 2:**
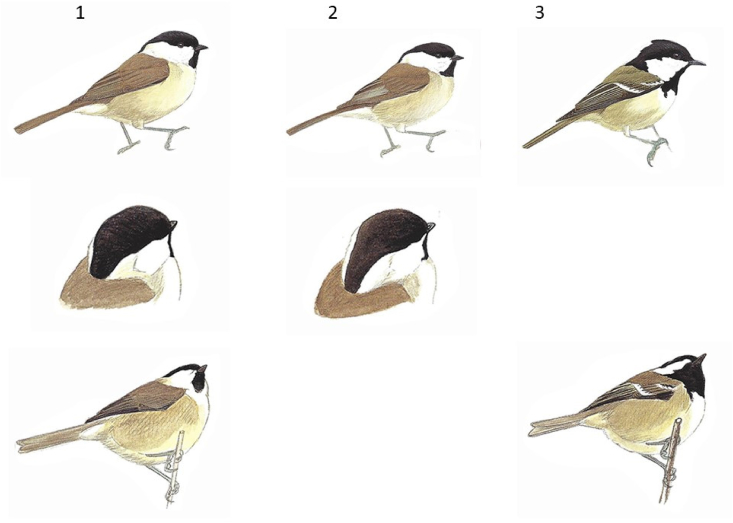
Example of a trial from the advanced bird naming test. Participants were shown pictures of two or three commonly confused species of birds and were asked to name the bird in positions one, two and three. Birds were portrayed from several angles to aid identification. In this example, the correct answers, from left to right, are: Marsh Tit, Willow Tit and Coal Tit. This image is reproduced with the permission of Bloomsbury. (For interpretation of the references to colour in this figure legend, the reader is referred to the web version of this article.)

A challenging test of bird naming was developed, using pictures of similar species that are commonly confused, for example Meadow and Tree Pipits or Marsh, Willow and Coal Tits (see Fig. 2). The test consisted of 14 trials where similar species of bird were presented alongside one another from various angles to facilitate identification. On each trial two or three birds were presented, depending on whether two or three species are easily confused. Participants were required to name the species presented in positions one, two and, if relevant, three. The maximum possible score was 30.

### 2.4 Statistical tests

Crawford and Garthwaite, (2005) modified t-statistic, run using the ‘singlims’ procedure, was used to establish whether SA was significantly impaired relative to controls on each test. The revised standardised difference test (Crawford and Garthwaite, 2005) was used to investigate whether the difference in SA's performance on separate tasks (e.g. test X vs. test Y) was significantly greater than that observed in the control group. All *p* values are one-tailed unless otherwise stated.

## 3 Results

### 3.1 Background neuropsychological assessment

SA's neuropsychological assessment showed intact performance on tasks of executive function, which contrasted with difficulties with verbal episodic memory and naming of visual stimuli, suggestive of a mild weakness in language/semantic memory (see Table 1). This is further indicated by his WAIS-IV Vocabulary performance. Although his score on this test was within the average range, when one considers his National Adult Reading Test and WAIS-IV Matrix Reasoning scores, his WAIS-IV Vocabulary performance is likely to reflect a subtle decline. The mild nature of his weaknesses in language/semantic memory may be explained by his predominantly right-sided pathology. Control participants did not significantly differ to SA on the WAIS-IV Vocabulary and Matric Reasoning subtests (see Table 2).

### 3.2 Basic auditory perception

SA's performance on the auditory perceptual discrimination test did not differ to that of controls (SA = 10/10, *M* = 9.80/10, *SD* = 0.42)*, t* (9) = 0.45, *p* = .33.

### 3.3 Tests of visual and auditory non-expert knowledge

**Fig. 3:**
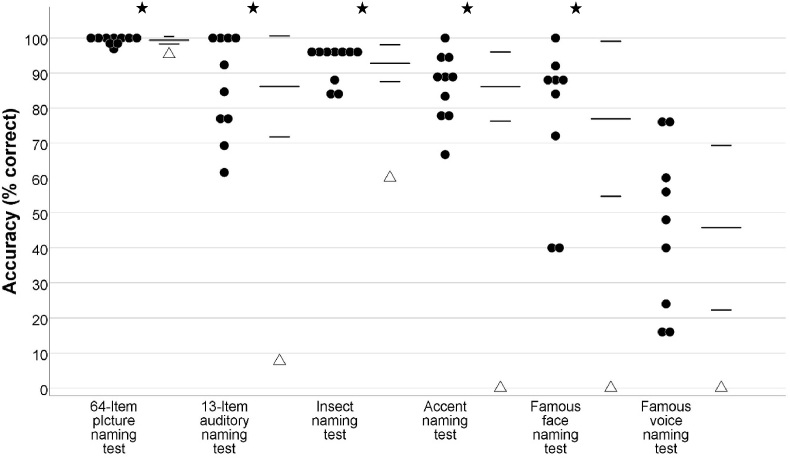
SA's performance on naming tests of non-expert knowledge, relative to that of controls. SA's scores are displayed as triangles and scores of individual healthy controls are displayed as circles. For each test, the healthy control group mean is shown by long horizontal line and one standard deviation is shown by shorter horizontal lines. Stars indicate a score significantly different to that of the control group. In the control group *N* = 10, except for the famous face and voice naming tests, where *N* = 9.

On tests of general (non-expert) object naming, SA was impaired for both pictures and sounds, demonstrated by significantly worse performance relative to controls on both the 64-item picture naming test (SA = 61/64, *M* = 63.60/64, *SD* = 0.70), *t* (9) = −3.54 *p* < .01, and 13-Item auditory naming test (SA = 1/13, *M* = 11.20/13, *SD* = 1.87), *t* (9) = −5.20 *p* < .001 (see Fig. 3). He appeared to be more severely impaired on the latter test, only correctly naming the sound of a cow, whereas his naming of the same 13 items from vision was flawless. For the 13 items that were presented as both pictures and sounds, a two-tailed revised standardised difference test confirmed that the difference between his performance on the two tasks was significantly greater than that observed in the control group, *t* (9) = 5.17, *p* < 001. On tests requiring non-expert within-category discrimination, SA's performance was significantly lower than that of controls on the insect naming test (SA = 15/25, *M* = 23.20/25, *SD* = 1.32), *t* (9) = −5.92, *p* < .001, and accent naming test (SA = 0/9, *M* = 7.75/9, *SD* = 0.89), *t* (9) = −8.30, *p* < .0001. His accent naming performance was weaker than his insect naming performance and a two-tailed revised standardised difference test showed that the difference between his performance on the two tasks was significantly greater than that observed in the control group, *t* (9) = 2.89, *p* < .05^3^

SA failed to score on the famous face and voice naming test. His performance fell significantly below that of controls for naming of famous faces (SA = 0/25, *M* = 19.22/25, *SD* = 5.54), *t* (8) = −3.29, *p* < .05, and there was a trend towards performance below that of controls for naming famous voices (SA = 0/25, *M* = 11.44/25, *SD* = 5.88), *t* (8) = −1.85, *p* = .05.

### 3.4 Tests of visual and auditory expert knowledge

**Fig. 4:**
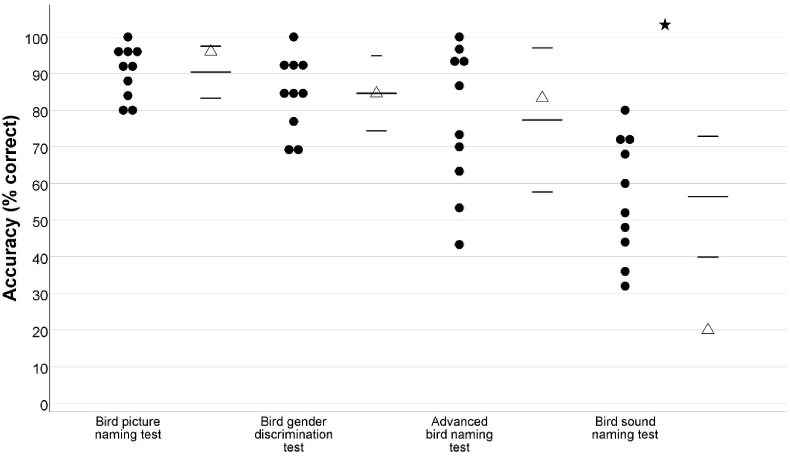
SA's performance on bird naming tests, relative to that of controls. SA's scores are displayed as triangles and scores of individual healthy controls are displayed as circles. For each test, the healthy control group mean is shown by long horizontal line and one standard deviation is shown by shorter horizontal lines. Significant differences are starred. In the control group *N* = 10.

On the bird picture and sound naming test, SA demonstrated intact naming of birds from pictures (SA = 24/25, *M* = 22.60/25, *SD* = 1.78), t (9) = 0.75, p = .24, and impaired naming of birds from sounds (SA = 5/25, *M* = 14.10/25, *SD* = 4.12), t (9) = −2.11, p < .05 (see Fig. 4). A two-tailed revised standardised difference test showed that the difference between his performance on the two tasks was significantly greater than that observed in the control group, *t* (9) = 2.30, *p* < .05 (see Fig. 4). SA's performance on individual items on these tests is presented in Supplementary Table 4.

SA's comments suggested intact knowledge of visually identifying characteristics e.g. when the colour of a bird's beak could be used to identify a bird's gender. Further investigation using a more challenging test of visual bird gender discrimination, showed that SA's visual bird discrimination performance did not differ from that of controls (SA = 11/13, *M* = 11/13, *SD* = 1.33), *t* (9) = 0, *p* = .50. He also demonstrated intact ability to name species of birds that are commonly confused, as his performance did not differ from that of the healthy bird experts on the advanced bird naming test (SA = 25/30, *M* = 23.20/30, *SD* = 5.90), *t* (9) = 0.29, *p* = .39.

Further analysis of SA's performance on individual items from the bird picture and sound naming test was undertaken to investigate whether his differential performance on visual vs. auditory bird naming tests could simply be explained by familiarity. Although he correctly names some well-known bird sounds (e.g. a cuckoo) he failed to name other well-known bird sounds (e.g. a dove). We split items on the bird picture and bird sound naming tests into those named most accurately and least accurately by the control group. For the bird picture naming test, there was no significant difference in performance between SA and controls for the pictures named most accurately by the control group (SA = 15/15, M = 15/15, SD = 0) or for the items named least accurately by the control group (SA = 9/10, M = 7.60/10, SD = 1.78, t (9) = 0.75, p = .24). In contrast, there was a significant difference in performance between SA and controls for the bird sounds named most accurately by the control group (SA = 5/12, M = 10.4/12, SD = 1.71, t (9) = −3.011, p < .01) and for the items named least accurately by the control group SA failed to score (SA = 0/13, M = 3.7/13, SD = 2.75). This suggests that SA's differential performance on visual vs. auditory bird naming tests is unlikely to be fully explained by familiarity effects.

### 3.5 Comparison between performance on tests of expert vs. non-expert knowledge

**Fig. 5:**
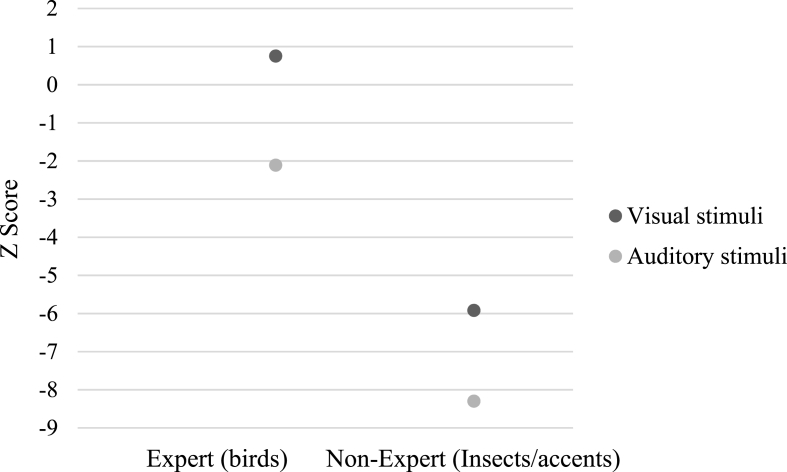
SA's performance on the bird picture and sound naming tests, the insect naming test and the accent naming test.

SA's performance was intact on the bird picture naming task but impaired on the insect naming task and a two-tailed revised standardised difference test showed that the difference between his performance on these tasks was significantly greater than that observed in the control group, *t* (9) = 4.91, *p* < .001. He also appeared to show weaker performance on the accent naming task than the bird sound naming task. A two-tailed revised standardised difference test showed the difference between his performance on these tasks was significantly greater than that observed in the control group, *t* (9) = 4.85, *p* < .01. SA's performance on these tests, relative to that of the control group, is presented in Fig. 5.

## 4 Discussion

The current study reports on SA, a bird ringing expert with predominantly right-sided SD like the report by Muhammed et al., (2018) another bird expert with predominantly left-sided SD, SA showed impaired auditory avian knowledge and intact visual avian knowledge. When considered in isolation, these findings could be taken as evidence that expertise may have differential protective effects upon auditory and visual knowledge. However, further systematic investigation revealed that this is not the most plausible explanation for these findings.

The current study furthered that of Muhammed et al. (2018), by conducting a detailed assessment of visual and auditory knowledge of both expert and non-expert categories. This revealed that SA showed a clear modality specificity effect, with relatively weaker naming of items when presented auditorily vs. visually, that was not restricted to his expert category. This pattern was observed on tasks of basic-level naming (64-item picture naming test vs. 13-item auditory naming task), within-category non-expert naming (insect naming test vs. accent naming test) and within-category expert-naming (bird picture naming test vs. bird sound naming test). While the healthy control group was also weaker on auditor vs. visual tests, SA's difference in performance between auditory vs. visual tests was significantly greater than that observed in the control group. As we will later go on to discuss, no difference was observed between his performance naming famous faces and voices, as his performance was at floor on these tasks. Although the current investigation does not have a direct bearing on theories relating to the existence of an integrative multimodal or amodal ‘hub’ (Patterson and Lambon Ralph, 2016) this striking discrepancy supports previous claims that semantic input channels via different sensory modalities are to some extent separable and differentially vulnerable in semantic dementia (Butler et al., 2009; Muhammed et al., 2018).

SA's performance is suggestive of the typical pattern observed in SD (Rogers and McClelland, 2004) of hierarchical loss of knowledge: deterioration of within-category knowledge, followed by degradation of knowledge of superordinate and generic features (see Fig. 3). It should be acknowledged, however, that we cannot make strong inferences about this pattern, given that the data are not longitudinal and the performance on the control group suggests that the tests of famous faces and voices were particularly challenging and the tests of superordinate knowledge approached ceiling levels. Nevertheless, when the current findings of relative vulnerability of auditory vs. visual knowledge are taken together with previous evidence of hierarchical loss of knowledge, tests of fine-grained auditory knowledge may be particularly sensitive to the early symptoms of SD.

In addition to an effect of modality-specificity, the current findings demonstrated an independent effect of expertise: SA's performance was relatively intact when asked to name objects from his expert vs. non-expert category. This was the case for both visual (bird picture naming vs. insect naming) and auditory (bird song naming vs. accent naming) modalities. Such an effect is unlikely to be explained by a confound associated with the type of non-expert categories that were selected, as his knowledge of birds was compared to his knowledge of other categories of living things, requiring a similar level of within-category discrimination. Thus, it is likely that the present findings reflect a genuine effect of expertise. This demonstrates, for the first time, that auditory and visual knowledge are modulated by expertise to a similar extent.

What is less clear however, is the nature of this ‘expertise’ effect. Parallel distributed processing (PDP) models of semantic cognition (e.g. Rogers and McClelland, 2004), suggest that domain-specific expertise increases the differentiation of specific-level concepts. For example, studies of unconstrained sorting show that experts divide objects in their domain of expertise into a greater number of groups than do novices (Boster and Johnson, 1989). In a simple computer model of semantic memory, Rogers and McClelland (2004) presented types of either fish or bird more frequently than other items during training. They found that their model differentiated subordinate items to a greater degree in the expert domain and that objects in the expert domain were on average further apart from one another in representation space than were items from non-expert categories. According to their model, even in the context of global semantic deterioration in SD, expert concepts should be relatively preserved because they are more differentiated. Thus, in bird experts, highly similar specific-level concepts, such as Marsh Tit, Willow Tit and Coal Tit, are represented by more distinct patterns of activation than they are in non-experts. Whilst Rogers and McClelland (2004) demonstrated that exposure is a key mechanism underlying the acquisition of domain-specific expertise, they argued that experts and novices may not necessarily receive exposure to exactly the same kinds of information. For example, bird experts typically focus on distinguishing visual markings of particular species of birds, while bird novices may have little awareness of many of these distinguishing markings (Tanaka and Taylor, 1991). One particularly important factor may be that knowledge of avian characteristics tends to be acquired through deliberate, structured learning. In fact, the explicit goal of such learning is typically to acquire knowledge of key identifying characteristics. This suggestion is supported by SA's comments during testing, where on several occasions, unprompted, he verbally reported the characteristic that he used to aid identification. Rogers and McClelland (2004) suggest that, in comparison to novices, bird-experts’ greater experience weighted toward information that distinguishes particular birds does not simply accelerate learning about object properties. Instead, bird experts come to represent such items differently—differentiating them to a greater degree, and consequently devoting a broader semantic subspace to their representation.

It is important to consider that one possible interpretation is that frequency effects could also explain the modality effects observed here and in the study by Muhammed et al. (2018). Given the hierarchical nature of semantic knowledge, it could be argued that humans are predisposed to favour or rely more on visual over auditory information, resulting in auditory knowledge being more vulnerable to deterioration. There are, however, compelling reasons why this is unlikely to be the case. For example, while in some situations more relevant information may be gained from visual over auditory information, the exact reverse is true in other situations. Indeed, in the case of music, auditory information is more dominant than visual. It could also be argued that the items included in the 64-item picture naming task vs. 13-item auditory naming task and the bird picture vs. bird sound naming tests may be more familiar visually than auditory. Indeed, SA only correctly named the sound of a cow on the 13-item auditory naming test, which one may suggest is because this item has high familiarity. However, he failed to name sounds which are also highly familiar, including that of a dog. Indeed, six of the sounds that he did not name had a higher word frequency than that of a cow (see Supplementary Table 1). Similarly, although some of the items that he correctly named on the bird sound naming test were well-known, such as a cuckoo, he failed to name other highly familiar birds, such as a collared dove.

Strikingly, SA's performance naming famous faces and voices was at floor. This contrasts with BA's (Muhammed et al., 2018) intact performance on tasks of famous face and voice knowledge. One explanation for this could be anatomical, as SA's damage was predominantly right-sided, whereas BA's was predominantly left-sided. Indeed, it is well established that impairments in famous face and voice recognition vs. famous name recognition are typically associated with atrophy predominantly affecting the right vs. left ATL (Gainotti, 2013, 2014; Snowden et al., 2004). An additional factor to consider is that, as Muhammed et al. (2018) acknowledge, BA's performance may be explained by differences in the task demands used to assess different categories of knowledge. Whereas, SA had relatively intact verbal semantics, BA had impaired verbal semantics, perhaps associated with his predominantly left-sided damage. Consequently, while it was possible to assess SA's knowledge of different categories using naming tasks, BA's avian semantic knowledge was assessed via forced choice decisions on associated semantic characteristics, such as habitat, whereas his person-based semantic knowledge was assessed by forced-choice familiarity decisions.

While the current findings extend understanding of the cognitive organisation of auditory semantics, several limitations must be acknowledged. All tasks required participants to respond by naming stimuli, as it was critical to ensure that a uniform response procedure was used across tasks. However, as SA's exhibited a mild weakness on a test of naming during his neuropsychological assessment, it is possible that this difficulty exacerbated the observed effects. A further challenge inherent in any attempt to draw comparisons between the integrity of semantic knowledge in different domains and across modalities is the selection of well-matched categories. For visual knowledge, insects were selected as a category against which knowledge of birds can be compared: both comprise exemplars of living organisms but do not include unique exemplars. Although we must concede that these categories are not perfectly matched, as there is arguably greater heterogeneity within the category of insects than within the category of birds. An alternative would have been to use dog breeds as a non-expert category of visual knowledge. However, for the purposes of the current study, this would have been problematic, as it is likely that knowledge of dog breeds is closely related to ownership of a dog, which could have confounded the results. For example, dog owners are often recommended to actively gain expertise in dog breeds prior to purchasing a dog. While it was feasible to recruit an adequate sample of control participants matched for years of expertise with birds, recruiting a control sample that was also matched for prior experience with dogs would have limited our ability to recruit a sufficiently large control sample. For auditory knowledge, we reasoned that accents provide a suitable category against which to measure knowledge of birds: again, both comprise exemplars of living organisms but do not include unique exemplars. However, we acknowledge that it would have been possible to use alternative categories, although this would not have been without limitations. Musical melodies, for example, would have provided a greater number of items, although this would have introduced the potential confound of contrasting sounds made from biological organisms and instruments.

We have shown for the first time that, while auditory knowledge may be more vulnerable than visual knowledge, expertise effects are similar across visual and auditory domains. While auditory semantic knowledge remains relatively poorly understood, the current findings illustrate how systematic investigations of single-cases can shed light on the principles underlying its organisation.

## Funding

CRB is supported by a Medical Research Council Clinician Scientist Fellowship (MR/K010395/1).

## CRediT authorship contribution statement

**J.A. Mole:** Conceptualization, Methodology, Investigation, Writing - original draft, Writing - review & editing, Supervision. **I.W. Baker:** Conceptualization, Methodology, Resources. **J.M. Ottley Munoz:** Investigation. **M. Danby:** Methodology. **J.D. Warren:** Writing - review & editing. **C.R. Butler:** Conceptualization, Methodology, Writing - original draft, Writing - review & editing, Funding acquisition, Resources, Supervision.

## Footnotes

1 Bird ringing involves the attachment of tags to the leg or wing of wild birds, undertaken so that individual birds can be identified and tracked to help monitor bird population and movement.

2 Data were missing from two participants on the accent naming test, so two additional age and education matched male control subjects were tested on this task.

3 As two participants in the accent naming test did not complete the insect naming test and vice versa, correlations used for the revised standardised difference test are based on the eight participants who completed both tasks. This also applies to the Revised Standardised Difference Test used to compare performance between the accent naming test and the bird sound naming test.
