# Supplementary material for: Avian agnosia: A window into auditory semantics
Source: Neuropsychologia. 2019 Nov;134:107219. doi: 10.1016/j.neuropsychologia.2019.107219 (PMC6891886; doi:10.1016/j.neuropsychologia.2019.107219)
Supplement: Multimedia component 1 [file mmc1.docx]

*Supplementary table 1. Items included in the 64-item picture naming task and 13-item auditory naming task*

| **Item** | **Word Frequency** | **Included in 64-item picture naming task** | **Included in 13-Item auditory naming task** |
| --- | --- | --- | --- |
| Helicopter | 10.8872 | X | X |
| Mouse | 8.44797 | X |  |
| Toaster | 0.65442 | X |  |
| Strawberry | 2.97464 | X |  |
| Suitcase | 12.9694 | X |  |
| Cat | 43.3107 | X | X |
| Bicycle | 18.4428 | X |  |
| Apple | 18.4428 | X |  |
| Rabbit | 11.0062 | X |  |
| Sledge | 1.13036 | X |  |
| Dustbin | 2.32022 | X |  |
| Frog | 3.62906 | X | X |
| Tomato | 7.25812 | X |  |
| Lorry | 7.61507 | X | X |
| Cow | 23.2617 | X | X |
| Watering can | N/A | X |  |
| Pineapple | 2.32022 | X |  |
| Bus | 65.0851 | X | X |
| Stool | 9.34036 | X |  |
| Dog | 73.2356 | X | X |
| Cherry | 5.7113 | X |  |
| Basket | 18.9187 | X |  |
| Train | 74.4254 | X |  |
| Squirrel | 3.74804 | X |  |
| Pear | 2.55819 | X |  |
| Horse | 87.8708 | X | X |
| Motorbike | 1.30884 | X |  |
| Banana | 4.28348 | X |  |
| Barrell | 14.8732 | X |  |
| Plane | 46.0474 | X | X |
| Orange | 28.973 | X |  |
| Piano | 26.6528 | X |  |
| Tortoise | 4.22399 | X |  |
| Pliers | 1.54681 | X |  |
| Key | 73.9495 | X |  |
| Penguin | 3.56957 | X |  |
| Axe | 7.49609 | X |  |
| Monkey | 9.28087 | X | X |
| Toothbrush | 1.72529 | X |  |
| Eagle | 7.55558 | X |  |
| Saw | 395.984 | X |  |
| Rhino | 0.237971 | X |  |
| Plug | 6.90116 | X |  |
| Chicken | 31.1147 | X | X |
| Spanner | 0.773406 | X |  |
| Kangaroo | 2.02275 | X |  |
| Glass | 130.408 | X |  |
| Duck | 9.8758 | X |  |
| Scissors | 4.52145 | X |  |
| Camel | 8.68594 | X |  |
| Envelope | 19.4541 | X |  |
| Owl | 3.21261 | X |  |
| Paintbrush | 0.594928 | X |  |
| Tiger | 9.28087 | X | X |
| Comb | 5.7113 | X |  |
| Swan | 5.17587 | X |  |
| Screwdriver | 2.91515 | X |  |
| Elephant | 12.8504 | X | X |
| Candle | 8.03152 | X |  |
| Ostrich | 1.72529 | X |  |
| Alligator | 1.42783 | X |  |
| Brush | 18.3833 | X |  |
| Peacock | 2.97464 | X |  |
| Hammer | 11.8986 | X |  |

**Note:** Word frequency was obtained from the Celex database, extracted through the MCWord online programme (www. neuro.mcw.edu/mcword/).
