# Supplementary material for: Avian agnosia: A window into auditory semantics
Source: Neuropsychologia. 2019 Nov;134:107219. doi: 10.1016/j.neuropsychologia.2019.107219 (PMC6891886; doi:10.1016/j.neuropsychologia.2019.107219)
Supplement: Multimedia component 2 [file mmc2.docx]

*Supplementary table 2. Scoring criteria for the accent naming test*

Participants were awarded one point if they correctly named the country of origin and half a point if their response was partially correct, defined as an accent that is commonly confused with the correct accent. Incorrect answers were awarded zero points. Answers were considered partially correct and awarded half a point when participants’ guesses were any of the following:

- New Zealand, when the accent was from Australia.
- Canada, when the accent was from the United States of America.
- Countries formally within the Union of the Soviet Socialist Republic, when the accent was from Russia.
- Confusion between countries within the United Kingdom (England, Scotland, Wales and Ireland).

In the control group, half points for partially correct answers were awarded infrequently: 83.33% of guesses were correct, 5.56% were partially correct and 11.11% were incorrect.
