# Supplementary material for: Avian agnosia: A window into auditory semantics
Source: Neuropsychologia. 2019 Nov;134:107219. doi: 10.1016/j.neuropsychologia.2019.107219 (PMC6891886; doi:10.1016/j.neuropsychologia.2019.107219)
Supplement: Multimedia component 3 [file mmc3.docx]

*Supplementary table 3. Items included in the famous face and voice naming test*

| **Famous person** |
| --- |
| Elvis Presley |
| Tony Blair |
| Margaret Thatcher |
| Paul McCartney |
| Tom Jones |
| Donald Trump |
| Simon Cowell |
| Boris Johnson |
| Sean Connery |
| Tom Cruise |
| Noel Edmonds |
| George Clooney |
| Michael Jackson |
| Terry Wogan |
| Dawn French |
| Sue Barker |
| Ronnie Corbett |
| George Bush |
| Eddie Izzard |
| Jo Brand |
| Michael Caine |
| Jonathon Ross |
| Billy Connolly |
| Barbara Windsor |
| Cher |
