# Supplementary material for: Avian agnosia: A window into auditory semantics
Source: Neuropsychologia. 2019 Nov;134:107219. doi: 10.1016/j.neuropsychologia.2019.107219 (PMC6891886; doi:10.1016/j.neuropsychologia.2019.107219)
Supplement: Multimedia component 4 [file mmc4.docx]

*Supplementary table 4. Items included in the bird picture and sound naming test*

| **Item** | **Stimuli** | **Stimuli** | **Accuracy** |
| --- | --- | --- | --- |
| 1 | Green woodpecker | Picture | Correct |
| 2 | Wigeon | Picture | Correct |
| 3 | Green Sandpiper | Picture | Correct |
| 4 | Mallard | Sound | Incorrect |
| 5 | Red Kite | Picture | Correct |
| 6 | Lapwing | Picture | Correct |
| 7 | Woodcock | Sound | Incorrect |
| 8 | Kingfisher | Sound | Incorrect |
| 9 | Kestrel | Sound | Incorrect |
| 10 | Blue Tit | Picture | Correct |
| 11 | Buzzard | Sound | Incorrect |
| 12 | Grey Heron | Sound | Incorrect |
| 13 | Carrion Crow | Picture | Correct |
| 14 | Mute Swan | Picture | Correct |
| 15 | Willow Warbler | Picture | Incorrect |
| 16 | Red-legged partridge | Picture | Correct |
| 17 | Tufted Duck | Sound | Incorrect |
| 18 | Jackdaw | Sound | Incorrect |
| 19 | Chiffchaff | Sound | Correct |
| 20 | Canada Goose | Picture | Correct |
| 21 | Golden Plover | Sound | Incorrect |
| 22 | Collard Dove | Picture | Correct |
| 23 | Tawney owl | Sound | Correct |
| 24 | Sparrowhawk | Picture | Correct |
| 25 | Cuckoo | Sound | Correct |
| 26 | Carrion Crow | Sound | Correct |
| 27 | Cuckoo | Picture | Correct |
| 28 | Sparrowhawk | Sound | Incorrect |
| 29 | Canada Goose | Sound | Correct |
| 30 | Kingfisher | Picture | Correct |
| 31 | Golden Plover | Picture | Correct |
| 32 | Kestrel | Picture | Correct |
| 33 | Mute Swan | Sound | Incorrect |
| 34 | Chiffchaff | Picture | Correct |
| 35 | Woodcock | Picture | Correct |
| 36 | Jackdaw | Picture | Correct |
| 37 | Lapwing | Sound | Incorrect |
| 38 | Mallard | Picture | Correct |
| 39 | Red Kite | Sound | Incorrect |
| 40 | Collared Dove | Sound | Incorrect |
| 41 | Grey Heron | Picture | Correct |
| 42 | Wigeon | Sound | Incorrect |
| 43 | Tawney Owl | Picture | Correct |
| 44 | Tufted Duck | Picture | Correct |
| 45 | Green Woodpecker | Sound | Incorrect |
| 46 | Willow Warbler | Sound | Incorrect |
| 47 | Red-legged Partridge | Sound | Incorrect |
| 48 | Buzzard | Picture | Correct |
| 49 | Blue Tit | Sound | Incorrect |
| 50 | Green Sandpiper | Sound | Incorrect |
